# A survey and evaluation of population-based screening for gastric cancer

**DOI:** 10.7497/j.issn.2095-3941.2013.02.002

**Published:** 2013-06

**Authors:** Yuan Yuan

**Affiliations:** Tumor Etiology and Screening Department of Cancer Institute and General Surgery, The First Affiliated Hospital of China Medical University, and Key Laboratory of Cancer Etiology and Prevention, Liaoning Provincial Education Department, Shenyang 110001, China

**Keywords:** Gastric cancer, screening, endoscopy, pepsinogen, evaluation studies

## Abstract

Screening and early diagnosis of gastric cancer play important roles in reducing the mortality of gastric cancer. A vast amount of study data on gastric cancer screening and early diagnosis has been accumulated in and out of China in the past decades. The practice of gastric cancer screening has also been efficiently carried out in different countries and regions. However, no widely accepted principle of population screening for gastric cancer has been developed yet. Screening for gastric cancer requires extensive exploration both theoretically and practically. This article focuses on the method and program of gastric cancer screening based on population. Moreover, the current situation of gastric cancer screening and its evaluation are evaluated.

## Introduction

Gastric cancer is the second most common cause of death in all types of malignant tumors, preceded only by lung cancer^1^. According to the data released by the Ministry of Health, the mortality of gastric cancer in China comes third after lung cancer and liver cancer^2^. Early detection, diagnosis, and treatment can effectively reduce the mortality of gastric cancer. The perfect execution of the “Three Early” concept involves the adoption of a screening practice based on an effective screening method and program.

Screening for gastric cancer consists of two models: population-based or mass screening, which is organized by governments or institutions across the entire population or the target population of a country, and opportunistic or individual screening, which is the passive screening of individuals with high risk factors in the course of medical treatment. For decades, numerous studies on gastric cancer screening and early detection have been reported throughout the world, and successful gastric cancer screening practices have been carried out in different countries and regions. Nevertheless, no widely accepted principle of population screening for gastric cancer has been developed yet. Screening for gastric cancer requires extensive exploration both theoretically and practically. This article presents a general survey and evaluation of population-based gastric cancer screening in China and abroad.

## Methods and programs for gastric cancer screening

### Methods for gastric cancer screening

The technological methods for gastric cancer screening include traditional barium double-contrast radiography and endoscopy combined with biopsy as well as serum biomarker detection.

#### Barium double-contrast radiography

Barium double-contrast radiography aims to show clearly the fine structure of gastric mucosa through double-contrast, mucosa, permeation, and press images. This method was created by the Japanese scholar Shirakabe Hikoo in 1950^3^. Since the end of the 1970s, Japan has gradually established a nationwide government-sponsored mass screening program. The program adopts indirect barium double-contrast radiography as the prescreening method, and then conducts X-ray detection or direct endoscopy for the at-risk population above the age of 40. Five million cases of gastric cancer are detected annually. The detection rate of gastric cancer is 0.12%, and the sensitivity and specificity are 82.4% and 77.2%, respectively^4^. However, barium-endoscopy detection in Japan costs approximately 7,000 USD to discover one case of gastric cancer. In addition, a randomized controlled trial for barium-endoscopy detection has not been adopted, preventing us from evaluating the effects of lead-time bias, length bias, and self-choice bias on survival rate, or reaching a scientific conclusion on the effect of this method. Therefore, barium-endoscopy detection has not been recommended to other countries by the World Health Organization (WHO) and the International Cancer Alliance (ICA).

#### Endoscopy

Endoscopy aims to achieve detection of all parts of the stomach from multiple angles under direct vision. The improvement of endoscopic equipment has significantly improved the sensitivity, specificity, accuracy, and security of endoscopic technology. Through direct vision combined with biopsy, a final pathologic diagnosis can be made. Currently, some severe precancerous lesions and early gastric cancers (EGC) can be detected and cured by endoscopy. In opportunistic or individual screening, endoscopy is used as a first-line inspection method. However, in large-scale population screening, endoscopy is usually used for diagnostic detection after the prescreening^5^. Problems such as cost, security, compliance, and technical training are encountered in the direct application of endoscopy for mass screening. Thus, this method has not been recommended in a population census by the WHO.

#### Serum biomarker detection

Serum biomarkers currently used for gastric cancer screening include differentiated products of the gastric mucosa such as pepsinogen and gastrin; gastric-cancer-associated antigens such as Mg7; protein antigens such as CA72-4, CA19-9, and CEA; and gastric pathogens such as *Helicobacter pylori (H. pylori)*. The specificity, sensitivity, costs, and implementing conditions of the aforementioned biomarkers are different.

##### Pepsinogen

Pepsinogens (PGs), including pepsinogen I (PGI) and pepsinogen II (PGII), are the precursors of specific functional enzymes of the gastric mucosa. PGI is secreted by the chief cell of the fundic gland, and PGII is secreted by the antral and pyloric gland cells. When the gastric mucosa is inflamed, the serum concentrations of PGI and PGII increase. Generally, the increase of PGII is more distinct than that of PGI, and so the PGI/II ratio is lower. In patients with gastric mucosal atrophy, the serum concentrations of PGI and PGII decrease with the development of atrophy and the disappearance of differentiated cells. Generally, PGI has a more obvious decrease than PGII, and so the PGI/II ratio is also lower. Low PGI, low PGI/II ratio, or low values of both are the valid flags used to judge the atrophic changes of gastric mucosa, as well as the warning flags used to identify high-risk individuals. Samloff established PG radio-immunity analysis in 1982^6^; since then, several studies have regarded it as the appropriate prescreening tool for gastric cancer. Oishi *et al.*^7^ reported that the combined detection of serum PGI and PGI/II ratio is a better index to predict the development of gastric cancer. After a 14-year follow up, the individuals with strongly positive serum PG had at least four times higher incidence of gastric cancer than those with negative serum PG. This association remained significant in individuals who were positive or negative for *H. pylori* antibody. Yuan *et al.*^8^ found that the sensitivity and specificity of the PG prescreening program drawn up by stratification based on age, gender, and other factors could be from 63% to 83% and from 51% to 76%, respectively. The prescreening for serum PG could identify the high-risk population before diagnostic detection and decrease endoscopic examination rate of target subjects to 50%. At present, serum PG detection has been applied to the gastric cancer screening of high-risk populations in Japan^9^, Finland^10^, China^11^^,^^12^, and other countries and regions.

##### Gastrin 17

Gastrin 17 (G17) is recommended for the identification of patients with gastric atrophy, particularly the extent of chronic atrophic gastritis (CAG). G17 concentration in serum or plasma depends on the intra-gastric acidity and the number of G cells in the antrum. G17 can detect CAG limited to the antrum (low G17 concentration) or the corpus (high G17 concentration) by combining with the serum PG assay. A Japanese study demonstrated that serum G17 and PG combined with gastric histological examination can identify populations that are at high risk for gastric cancer. Low PGI, low PGI/II ratio, and high G17 are proved to be factors leading to CAG of the corpus and the fundus of the stomach. For example, individuals may have a higher risk for developing gastric cancer if the lesser curve of the corpus is accompanied by intestinal metaplasia (IM). However, the concentration of serum G17 is only related to the distal stomach and increases prominently in gastric cancer. Therefore, the concentration of serum G17 cannot distinguish EGC from advanced gastric cancer, and the method cannot be used as an independent serological indicator of gastric cancer^13^.

##### Gastric cancer-associated antigen (GCAA)

MG7-Ag is a member of GCAA^14^. Nowadays, high-response immune-PCR technology to detect serum MG7-Ag has been developed^15^. Zhang *et al.*^16^ employed immune-PCR technology to conduct a double-blind detection of serum MG7-Ag among 2,710 people, and considered endoscopic biopsy with histopathological diagnosis as the evaluation standard. Results indicated that the sensitivity, specificity, and accuracy of serum MG7-Ag for gastric cancer detection reached 77.5%, 95.5%, and 73.1%, respectively. As a tumor biomarker, MG7-Ag has a significant application potential in the screening and detection of gastric cancer^17^. Jin *et al.*^18^ analyzed the expression difference of the serum MG7-Ag antigen in patients with gastric cancer before and after operation, patients with gastric precancerous diseases, healthy blood donors, and other cancer patients. They found the highest expression of serum MG7-Ag in preoperative gastric cancer patients. In the study, the expression level of serum MG7-Ag in gastric cancer was related to tumor differentiation and pathological stage. The analysis indicated that Mg7-Ag may be used as a non-invasive method for large-scale screening of a high-risk gastric cancer population.

##### Other protein antigens

Carbohydrate antigens CA72-4 and CA19-9, and carcinoembryonic antigen (CEA) are biomarkers related to gastrointestinal tumor. Studies have shown that the specificity and sensitivity of CA72-4 to detect gastric cancer are higher than those of CEA and CA19-9. The sensitivity of a combined assay with these three tumor markers is 76%, which is obviously higher than that of a single tumor marker^19^. Vascular endothelial growth factor (VEGF) is the strongest and most specific factor currently known to stimulate directly the proliferation effect of vascular endothelial cells. Studies have demonstrated that the concentration of serum VEGF in the gastric cancer group is obviously higher than that in the gastric ulcer group and the control group^20^. Tanaka *et al.* conducted research and proved that, on average, the serum hepatocyte growth factors (HGFs) of gastric cancer patients are considerably higher than those of healthy people. The positive rates of HGFs in early cases are higher than those of CEA and CA19-9. The serum HGFs level of gastric cancer patients with peripheral vessel invasion is significantly higher than that of patients without peripheral vessel invasion. Among the EGCs, the increase of serum HGFs is related to lymphatic metastasis. Thus, serum HGFs level may be a key tumor marker of gastric cancer^21^.

##### H. pylori

*H. pylori* infection is a major pathogenic factor in gastric cancer. A prospective study that included 1,526 cases in Japan reported that 36 cases (2.9%) among 1,246 people infected with *H. pylori* suffered from gastric cancer during the 10.6-year follow up. No new cases of gastric cancer were found in people with negative *H. pylori* infection. Serum detection for *H. pylori* antibody can be used as a screening method for populations with high risk of gastric cancer, and people who have *H. pylori* infection should be inspected further^22^. The Asian-Pacific Consensus Guidelines on Gastric Cancer Prevention demonstrated that *H. pylori* infection screening can reduce the risk of gastric cancer in the high-risk population^23^. The combined detection of serum *H. pylori* antibody and PG is an effective index to predict the occurrence of gastric cancer. A large-scale series of studies on 9,000 people in Japan showed that after follow-up for an average period of 4.7 years, serum PG indicated that the onset risk of gastric cancer for patients with atrophic gastritis is six to eight times higher than that for patients with normal serum PG concentration and negative *H. pylori* serology. Notably, individuals with negative *H. pylori* serology have a higher risk than those with positive serology, perhaps due to the absence of *H. pylori* when atrophy is severe. Male patients over 60 years old and with negative *H. pylori* serology have the highest average annual incidence (1.8%) of gastric cancer^24^.

#### Genetic biomarker

Hereditary diffuse gastric cancer (HDGC), a rare, inherited cancer syndrome with autosomal dominant mutation of CDH1, accounts for 1% to 3% of all gastric cancers. About 30% to 40% of the families in accordance with the clinical criteria for HDGC have a germline mutation of CDH1. The International Gastric Cancer Linkage Consortium provided the following standards for consenting to CDH1 molecular genetic testing: (1) the presence of two or more gastric cancer cases in the family, with at least one case histologically confirmed as diffuse gastric cancer, age <50 at diagnosis; (2) three or more diffuse gastric cancer cases are observed among first- or second-degree relatives, independent of age of onset; (3) an individual is diagnosed with diffuse gastric cancer before the age of 40; and (4) an individual or family member has suffered from diffuse gastric cancer and lobular breast cancer, age <50 at diagnosis. Any patient who meets the above requirements should consent to undergoing mutation detection of CDH1. Health care experts with experience in genetic testing can provide genetic counseling to these patients before and after detection^25^.

The development of an overwhelming majority of gastric cancer cases is actually not dependent on a key gene mutation. Numerous minor and predisposing genes may act together and participate in the development of gastric cancer. Individuals who carry predisposing genes may be susceptible to cancer. This susceptibility is mainly a consequence of the polymorphisms of predisposing genes, which does not directly cause the cancerization of cells and the development of tumors, but makes the individual susceptible to special environmental factors. When exposed to a similar inducement, the person with predisposing genes can easily suffer from cancers. Currently, predisposing genes related to the carcinogenesis of gastric cancer include (1) genes related to the metabolizing function of detoxification of carcinogens, such as cytochrome oxidase CYP2E1, glutathione S-transferase GSTM1, GSTT1, GSTP1, and acetyltransferase NAT1 and NAT2; (2) genes related to DNA synthesis and repair, such as methylenetetrahydrofolate reductase as well as ribonucleotide repair genes XRCC1 and XRCC5; (3) genes related to immunologic function, such as interleukin IL-1B, IL-8, and IL-10; (4) genes related to the differentiation functions of gastric mucosa, such as mucoprotein MUC1 and pepsinogen PGC; and (5) genes related to cell cycle regulation, such as p53 and p16^26^^-^^30^. Detecting the polymorphisms of the predisposing genes of gastric cancer is crucial to identify individuals at high risk for gastric cancer and to prevent cancer.

### Programs for gastric cancer screening

The key to the early detection of gastric cancer is to draw up a feasible program using the current screening methods. According to the Union for International Cancer Control (UICC) standard^31^, an effective screening program should meet the following criteria: (1) high sensitivity; (2) economical, practical, and low cost; (3) painless, non-invasive, and easy for the subject to accept; (4) simple and easy to operate; (5) large-scale applicability and periodic review; (6) ability to improve treatment rate and reduce tumor-specific mortality; and (7) unlikely to cause high psychological and economic burden even with a false-positive result. Dong *et al.*^32^ believed that a reasonable technological protocol should consist of a two-step procedure, including general prescreening and diagnostic screening, but not excluding the one-step procedure covering prescreening and diagnostic detection. Whether the latter is appropriate or not depends on performance appraisal and health economic assessment.

**Figure 1:**
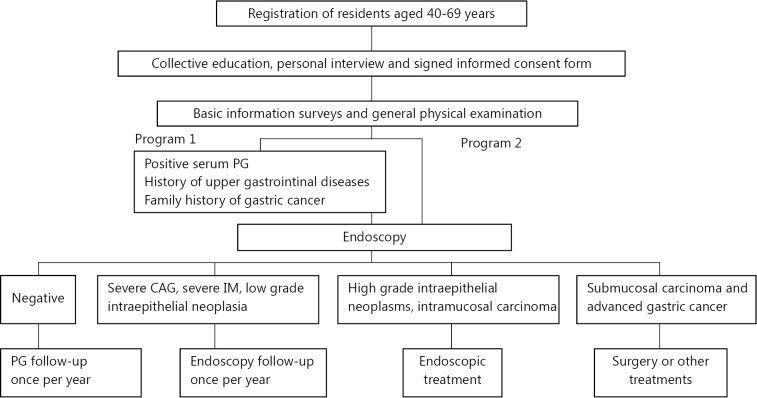
Flowchart of gastric cancer screening, early diagnosis, and treatment.

At present, no consensus exists for the gastric cancer screening program. Barium double-contrast radiography combined with endoscopy is a mature method that has been used in Japan for over 60 years. This method is complicated to use for population screening because it is expensive, has a certain degree of risk, and cannot be accepted easily by people. Therefore, it is not yet well applied across the world. For a long time, Chinese scholars have worked to determine the screening program for gastric cancer suitable to the national conditions in China. Some of the programs used were sequential screening, direct endoscopy, and PGI/II-endoscopy detection. Among these, PGI/II-endoscopy detection is the mass screening formula recommended and sponsored by the Central Financial Transfer Payment Projects since 2008 (**Figure 1**)^11^^-^^12^.

## The current situation of population screening for gastric cancer and its evaluation

### The current situation of population screening for gastric cancer in Asia

The incidence and mortality of gastric cancer differ significantly by region, population, and race distribution. Restrictions caused by social and economic conditions and health care systems lead to differences in the demand for and implementation of gastric cancer screening, prevention, and control from one country to another. At present, the East Asia region, particularly Japan, Korea, Singapore, China, and other countries with a high incidence of gastric cancer, has achieved tangible results from its screening strategies, as well as from the establishment of screening programs and intervention measures^13^. No nationwide screening of gastric cancer has been reported in the United States, Europe, and other areas with a low incidence of gastric cancer.

#### Japan

Japan is one of the countries with a high incidence of gastric cancer. It has achieved remarkable achievements in screening for gastric cancer. Since the beginning of the 1960s, a nationwide screening program using barium double-contrast radiography combined with endoscopy has been set up in Japan. The Japanese government encourages people over the age of 40 to undergo gastric cancer screening annually and subsidizes the cost of such screening. Any person who tests positive in barium meal screening undergoes further endoscopy detection. The census coverage is maintained within three to five million people each year. A total of 3,000 to 6,000 gastric cancer cases are detected annually. Among these cases, EGC accounts for approximately 50% to 70%. An estimated 50% of patients undergo resection or delamination of the gastric mucosa through endoscopy^33^.

Upper gastrointestinal endoscopy is another screening method used in Japan. The routine examination is applied to the opportunistic screening of clinical outpatients who do not participate in the national screening or decide to go to private clinics for health care. In the Japanese health insurance system, regardless of their individual symptoms, people can receive upper gastrointestinal endoscopy in clinics. They pay less than 30% of the examination cost, and the remaining cost is paid through government insurance. However, private health care clinics do not participate in the government insurance system; therefore, all of the expenses have to be paid by the patients. Only a small number of people undergo endoscopic tests in private clinics. The majority of endoscopies are carried out in clinics under the government insurance system, a situation that contributes significantly to the high detection rate of EGC in Japan^13^.

Barium-meal screening combined with endoscopy detection is the only program adopted by the national census of gastric cancer in Japan. However, only 20% of the qualified individuals participate in the screening. In 2002, about 4.3 million people participated in the gastric cancer screening organized by local governments. In 2010, 34.3% of the national population accepted the government-sponsored screening. In 2009, the detection rate of gastric cancer was only 8.8%^34^.

To boost the declining participation in the national screening program for gastric cancer and increase the detection rate, the Japanese government started to seek a new screening strategy that would encourage the adoption of a combined examination of serum PG and *H. pylori*. The combined examination could be used as an effective etiological prevention tool for high-risk populations and as a subsequent screening method for gastric cancer^34^. Since the 1990s, the detection of serum PG combined with endoscopy has been conducted in Japan to screen for gastric cancer. Miki K. *et al.*^35^ reported that among 101,892 Japanese, 125 cases of gastric cancer were detected using serum PG-endoscopy, with a detection rate of 0.12%. Among these cases, EGC accounted for 80%.

#### Other countries in Asia

Gastric cancer is one of the most common malignant tumors in Korea. It ranks the second among tumor-related causes of death. In 2002, Korea officially launched the gastric cancer national census. The direct upper gastrointestinal series or endoscopic detection (or a combination) was adopted to encourage Korean males and females above the age of 40 to undergo gastric cancer screening every two years. The detection rate of EGC was 20%; among the asymptomatic population, the rate was 74% to 78%, which was obviously higher than that for the symptomatic population (26% to 36%). The national gastric cancer census report of Korea in 2005 showed that the predicted detection rate of gastric cancer was about 0.12% (1,381 among 15 million people)^36^. Owing to the early diagnosis and treatment of gastric cancer, the 5-year survival rate of gastric cancer in Korea gradually increased from 40% during the 1990s to more than 60% at the beginning of this century^37^. Endoscopy is the screening method with the highest cost-effectiveness. This method has a high detection rate of gastric cancer but with relatively low cost. However, a randomized controlled trial has not been conducted yet to evaluate the mass screening of gastric cancer, and additional data are required to support the existing screening projects^13^.

Singapore has a moderate incidence of gastric cancer. The subjects of programs for gastric cancer screening are Chinese ≥50 years old with high-risk incidence, as well as Malaysians and Indians with moderate- and low-risk incidence. In 2003, the National Gastric Cancer Epidemiology and Molecular Genetics Program was established in Singapore to identify an optimum approach and cost-effectiveness ratio that would guide the local screening of gastric cancer. In 2004, Singapore launched a prospective cohort study. The government planned to recruit 4,000 high-risk patients older than 50 to participate in the systematic screening program for EGC. The clinical materials of the subjects were collected from the national database. According to a standard protocol, blood samples, gastric biopsy, gastric juice, and all types of biological samples were collected. Endoscopy was conducted to ensure follow-up for more than five years. The annual detection rate of gastric cancer or of precancerous lesion was 0.5%^38^. No national screening program for gastric cancer has been reported in Thailand, Malaysia, and other countries with a relatively low incidence of this disease.

#### China

Currently, no nationwide mass census of gastric cancer exists in China, though selective screenings of high-risk populations in areas with a high incidence of gastric cancer have been implemented. Ding *et al.*^39^ reported that a sequential screening (i.e., ultramicro-gastric juice analysis followed by endoscopy and histology as the final diagnosis) among 12,000 people in Muping County, Shandong Province, was conducted in the Seventh 5-year Plan. The detection rate of EGC was 47.2%, and the 5-year survival rate was 89%. You *et al.*^17^ reported that an endoscopy screening among 3,400 high-risk people in the 35 to 64 age range was conducted in Linqu County, Shandong Province. In the 10-year follow-up, 39,303 people underwent detection annually, and 85 cases of gastric cancer were found. The detection rate of EGC was 50% to 67%, and the total 5-year survival rate was 63.7%. Yuan *et al.*^40^ reported that a serum PG-endoscopy (two-step examination) screening was conducted in Zhuanghe County, Liaoning Province over a 10-year period. Among the 7,036 people who underwent two-step examinations, 69 cases of gastric cancer and 41 EGCs were detected. The detection rate of EGC was 56.8% to 64%. Since 2008, the two-step program has been adopted for population screening in areas at high risk for gastric cancer in China, including Zhuanghe, Linqu, and Wuwei (Ganshu Province), with support from the Central Financing Transfer Payment Project organized by the Ministry of Health. By the end of 2010, over 12,000 people had undergone the two-step screening, and the detection rate of EGC was 60% to 90%^17^^,^^40^. Zhang *et al.*^41^ conducted a 14-year serum (PG and *H. pylori*) follow-up among 1,501 high-risk residents in Zanhuang, Hebei Province. The incidence of gastric cancer was 56.0% among the high-risk population with abnormal serum PG and positive *H. pylori*.

### Evaluation of population screening for gastric cancer

According to the principle of evidence-based medicine, the evaluation of gastric cancer screening requires definite standards and methods^42^. The outcome index of performance assessment is the decrease of mortality rate. To perform health economic evaluation, the costs of early diagnosis and treatment of gastric cancer must be calculated, and the corresponding health benefits expressed by cost-effectiveness, cost utility, and cost benefit compared with the relative index without intervention. By combining the practical experience of early detection and treatment of gastric cancer in China, and by referring to the general principle of evaluation of cancer screening, Dong *et al.*^32^ proposed the feasibility of using the working index of early detection and treatment of gastric cancer (detection rate, early diagnosis rate, and cure rate) as the mid-term index. They further clarified the relationship between early detection cost index and health benefits, as well as the feasibility of indicators used in health economic evaluation, including evaluating indicators of the screening program, midterm indicators of performance appraisal, and health economic indicators. The aforementioned evaluation indicators may not be necessarily a substitute for classical performance assessment and health economic evaluation. However, they have primary judgment and path guidance functions in gastric cancer screening as well as have realistic implications on the promotion of early diagnosis and treatment of gastric cancer.

At present, reports related to evaluating the cost-effectiveness of mass screening for gastric cancer are extremely scarce. A study in Singapore shows that endoscopy for middle- and high-risk populations (such as Chinese males from 50 to 70 years old) once every two years remains a heavy burden for the medical insurance system of the country^43^. Zhou *et al.*^44^ adopted three types of basic methods of health economic evaluation, namely, cost-effect analysis, cost-benefit analysis, and cost-utility analysis, to conduct a health economic evaluation of two-step screening for gastric cancer. Results confirmed that the serum PG-endoscopy method is simple to operate and has a reasonable cost-effectiveness ratio. The compliance of subjects is also better. The method can avoid the potential hazard of nosocomial infection from endoscopy detection and reduce the diagnostic deviation resulting from the inspectors’ technology shortage. Two-step screening is currently in accordance with the national conditions in China; therefore, it deserves to be promoted and applied for gastric cancer screening in high-risk regions in the country.

## Problems and prospects

We conducted a general survey of the current situation and development trend of screening for gastric cancer, as well as investigated the early diagnosis and treatment of the disease in China and abroad, particularly in East Asian countries with a high incidence of gastric cancer. We find that the key problem encountered is establishing the optimal screening program and control strategy suitable for national conditions, including determining the screening scope, screening subjects, screening methods and programs, and intervention and treatment opportunities. The selection of a strategy for gastric cancer screening and control is restricted by factors such as economic condition and incidence and mortality of gastric cancer, as well as risk factors and screening tools for gastric cancer. Currently, China does not have the appropriate conditions to conduct a large-scale national gastric cancer census. Further research is necessary to improve the screening program, and efforts should be exerted to expand the early detection, diagnosis, and treatment of gastric cancer in high-risk regions.

The high detection rate of endoscopic technology has led to its widespread use in screening for gastric cancer. Specifically, endoscopy can detect superficial flat-type and non-ulcerative lesions missed by the traditional barium examination. However, the effectiveness of endoscopic technology largely depends on the skill of inspectors and the availability of endoscopic equipment. Thus, even in developed countries such as Japan, conducting large-scale population screening via endoscopy is unfeasible if experienced endoscopists are lacking. Endoscopy screening can increase the detection rate of EGC, but missed diagnoses can still occur. With the introduction of new endoscopic technologies such as chromoendoscopy, narrow-band imaging endoscopy, confocal endoscopy, and autofluorescence endoscopy, the availability and practicality of endoscopy will increase. Further studies are necessary to confirm the role of endoscopy in population screening for gastric cancer^13^.

Serum biomarker detection is non-invasive, easy to use, cost-effective, and has good compliance and better sensitivity and specificity. Thus, it can be used as a prescreening method for gastric cancer to detect high-risk populations. Based on the Japanese experience, PG is the most realistic and reliable serum marker for identifying high-risk populations with extensive gastric atrophy, and enables further endoscopic detection or other intensive surveillance methods^13^. However, the detection of serum PG has limitations: (1) it is used to detect atrophic gastritis, and is, therefore, suitable for screening intestinal-type gastric cancer; the effectiveness of serum PG in detection depends on the ratio of the intestinal-type gastric cancer patients to the test population; (2) the cut-off values of PG for mucosal atrophy and gastric cancer judgment vary in different regions; for example, in Japan, PGI less than 70 mg/L or PGI/II lower than 3 mg/L; can be used as the cut-off value to identify atrophic gastritis^35^. In China, PGI/II lower than 7 mg/L is used as the cut-off value to identify atrophic gastritis and gastric cancer risk^40^. Therefore, cut-off values of serum PG detection may be modified depending on the region, incidence of gastric cancer, population, and genetic background; (3) age of the patient and *H. pylori* infection may be directly related to gastric atrophy; these factors will affect the concentration of serum PG. Eradication therapy of *H. pylori* and proton pump inhibitors can change the concentration of serum PG to some extent. Although limitations exist, the concentration of serum PG has a high negative predictive value (e.g., it can identify individuals without gastric atrophy). Thus, individuals with negative detection results can avoid invasive screenings such as endoscopy^13^.

China is a developing country with a high incidence of gastric cancer. Therefore, determining a gastric cancer screening program that is suitable for the national conditions has practical significance. Serum biomarker detection exhibits superiority in population screening for gastric cancer despite several limitations. Improving the sensitivity and specificity of serum indicators and reducing the screening cost through a large-scale and multicenter study will have a significant impact on the early detection and treatment of gastric cancer in China. With the discovery of susceptibility genes for cancer, the accurate identification of high-risk individuals before malignancy will be realized, providing us new insights on the individual prevention of gastric cancer. Apart from having a higher risk of cancer, individuals with susceptibility genes for gastric cancer also have a lower age of onset than the healthy population, which is a new factor that challenges the use of traditional screening for gastric cancer. Further research must, therefore, be conducted and effective identification technology and screening files for high-risk individuals must be established. In China, the majority of the population lacks knowledge of EGC. Thus, a stronger cancer-prevention program and dissemination of related knowledge must be implemented to familiarize the population with proven-effective screening technology. Relatively healthy individuals should also be encouraged to participate voluntarily in the screening program for gastric cancer. These efforts will result in the maximum effectiveness of screening for gastric cancer in China.
